# From rammed earth to stone wall: Chronological insight into the settlement change of the Lower Xiajiadian culture

**DOI:** 10.1371/journal.pone.0273161

**Published:** 2022-08-22

**Authors:** Xiaojia Tang, Shui Shen, Xin Su

**Affiliations:** 1 School of History, Renmin University of China, Beijing, China; 2 School of Archaeology, University of Oxford, Oxford, United Kingdom; 3 Department of Anthropology, Harvard University, Cambridge, MA, United States of America; Washington University in Saint Louis, UNITED STATES

## Abstract

In this article, we investigate the chronological change of settlements of the Lower Xiajiadian (LXJD) culture in northeast China. On the basis of excavation data, two types of settlements can be identified based on the methods of site construction: earthen (rammed earth/mudbrick) settlements and stone-constructed settlements. After integrating and reanalyzing all published ^14^C radiocarbon data of different LXJD sites, we argue that there is a clear chronological difference between these two types of settlements. It is revealed by the OxCal model that settlements built with earthen structures are generally earlier than those constructed with stones, and the changes in settlement spatial distribution and constructive material largely happened after 1500 BC. By means of correlation analysis with other related archaeological evidence, we suggest that the underlying social dynamics that contributed to LXJD settlement changes can be explored through multiple prospects.

## 1. Introduction

**Fig 1:**
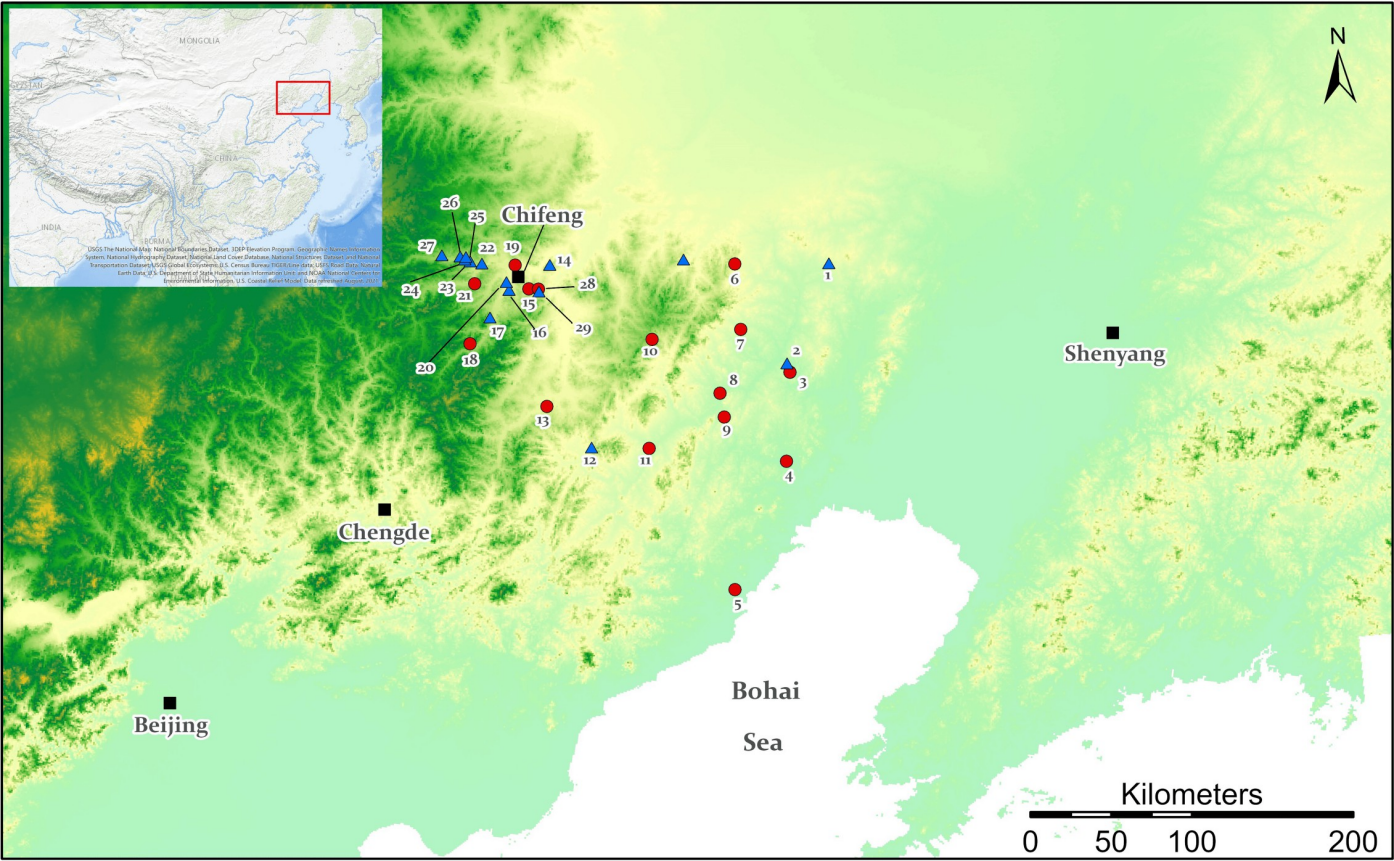
A map illustrating the distribution of excavated LXJD sites with radiocarbon dates. Map was produced in ArcGIS Pro. The digital elevation model was acquired from the Geospatial Data Cloud site, Computer Network Information Center, Chinese Academy of Sciences (http://www.gscloud.cn). 1. Pingdingshan 2. Kangjiatun 3. Xidachuan 4. Xidalizi 5. Xianlingsi 6. Dadianzi 7. Fengxia 8. Redianchang 9. Luoguodi 10. Kalaqinhedong 11. Shuiquan 12. Chengzishan 13. Xiaoyushulinzi 14. Danangou 15. Erdaojingzi 16. Dianjiangtai 17. Location 342 18. Dashanqian 19. Zhizhushan 20. Dongbajia 21. Dongshanzui 22. Xindian 23. Sanzuodian 24. Yantaishan 25. Shangjiyingfangzi 26. Xiliang 27. Kangjiawan 28. Gaojiataizi (Type A) 29. Gaojiataizi (Type B).

Dated to the second millennium BC, the Lower Xiajiadian (LXJD) culture (ca. 2000–1300 BC) was one of the earliest Bronze Age cultures rising at the northern border of modern China (Fig 1). Till today, more than 2000 stone and earthen walled sites labeled as the LXJD culture have been identified in southeastern Inner Mongolia and western Liaoning [1–8]. The discovery of these walled sites and associated archaeological materials in northeastern China marked the earliest episode of Bronze Age activities along China’s northern borders.

Among all the known LXJD locations, 27 sites have been well published with detailed reports (Tables 1 and 2). A total of 44 radiocarbon dates sampled from these 27 locations have been published throughout the years, including dates obtained from settlements, tombs, and stratigraphic sections. However, the archaeological materials of these settlements and radiocarbon dates have not been compiled and synthesized for an integrated discussion. In the following analysis, this paper first reassesses the location, landscape, and stratigraphic relationships of these 27 LXJD locations, and then with the support of Bayesian radiocarbon calibration, we reconstruct the chronology of LXJD settlement and discuss possible reasons for this sudden residential change in the mid-second millennium BC.

**Table 1:** Type A: Low-platform earthen sites.

Site	Site area (m^2^)	Excavated area (m^2^)	Reference
**Xidachuan**, Beipiao, Liaoning	-	1700	[24]
**Fengxia**, Beipiao, Liaoning	7,500	650	[12]
**Luoguodi**, Chaoyang, Liaoning	10,000	-	[25]
**Redianchang,** Chaoyang, Liaoning	20,000	575	[26]
**Shuiquan**, Jianping, Liaoning	20,000	2200	[14]
**Kalaqin Hedong**, Jianping, Liaoning	12,000	75	[27]
**Xianlingsi,** Xingcheng, Liaoning	17,550	800	[28]
**Xiaoyushulinzi**, Ningcheng, Liaoning	5,670	100	[29]
**Xidalizi**, Jinzhou, Liaoning	8,000	1,000	[30]
**Chengzishan**, Lingyuan, Liaoning	7,900	200	[31]
**Zhizhushan**, Chifeng, Inner Mongolia	20,000	100	[32]
**Dongshanzui**, Chifeng, Inner Mongolia	28,000	450	[13]
**Erdaojingzi**, Chifeng, Inner Mongolia	50,000	13,000	[19]
**Dashanqian**, Chifeng, Inner Mongolia	-	1550	[15, 16]
**Dadianzi**, Aohan, Inner Mongolia	17,000	220	[33]

**Table 2:** Type B: High hillfort stone sites.

Site	Site area (m^2^)	Excavated area (m^2^)	Reference
**Kangjiatun**, Beipiao, Liaoning	150,000	8,500	[17]
**Pingdingshan**, Fuxin, Liaoning	-	555	[34]
**Kangjiawan**, Chifeng, Inner Mongolia	-	1500	[35]
**Sanzuodian**, Chifeng, Inner Mongolia	11,600	9000	[36, 37]
**Location 34**, Chifeng, Inner Mongolia	-	-	[5]
**Dianjiangtai**, Chifeng, Inner Mongolia	-	-	[5]
**Dongbajia**, Chifeng, Inner Mongolia	22,400	-	[11]
**Shangjifangyingzi**, Chifeng, Inner Mongolia	-	2437	[18, 20]
**Xiliang**, Chifeng, Inner Mongolia	5,000	3000	[20, 38]
**Yantaishan**, Chifeng, Inner Mongolia	-	3000	[39]
**Xindian**, Chifeng, Inner Mongolia	10,000	-	[8]
**Danangou**, Chifeng, Inner Mongolia	7,000	-	[40]

## 2. Study area

The LXJD culture area is centered in southeastern Inner Mongolia and the western part of Liaoning province (Fig 1). The whole region is around 132,000 square kilometers. This is an area drained by the Xilamulun (Xar Moron)—Liao Rivers and its two tributaries, the Daling, and Xiaoling rivers. The uplift of the Great Khingan Mountains on the eastern edge of the Mongolian Plateau results in higher topography in the west (ca. 2800m a.s.l) than in the east (ca.500m a.s.l) in the east. Lying on the northern margin of the Pacific monsoon, the LXJD culture area is a transitional region between semi-humid and arid climates. The yearly average temperature in most parts of the LXJD area is between 0°C and 7°C with mean annual precipitation of ca. 250–600 mm [5].

## 3. Research background of the Lower Xiajiadian Settlements

The LXJD culture was first discovered in the 1930s, when Japanese archaeologists Hamada Kosaku and Mizuno Seiichi identified two types of archaeological remains at the Hongshanhou site [9], near present-day Chifeng city: Chifeng Phase I and Chifeng Phase II. It was the Phase II remains later confirmed as the LXJD culture. Chinese archaeologists started to explore the archaeology of this area during the Japanese occupation of World War II and soon after 1950. Three rounds of state-level archaeological survey projects (1956–1957, 1981–1985, and 2007–2011) and extensive regional surveys in the Chifeng region and Upper Daling River area have recorded the most exposed Lower Xiajiadian locations in the field and also revealed the geographical distribution of LXJD material remains and its distinctive cultural characteristics [5, 8, 10–21].

As two full-coverage regional surveys, both the Chifeng [5] and Upper Daling River (the West Liao River valley) [21] surveys take spatial distributions of collection units and surface scatters of artifacts as major proxies to delineate communities of different scales in the LXJD area. On top of these, estimates of population and community sizes are constructed. The estimated regional population densities are 33–66 person/km^2^ for the Chifeng survey area and 18–36 person/km^2^ in the Upper Daling region [22].

Both state-level and regional archaeological surveys confirmed that during all periods of LXJD occupation, there was a strong tendency to group settlements in clusters. The contrast in settlement pattern and variability in societal organization between the Chifeng region and western Liaoning has been noted in Drennan et al. [22], where the authors argued that the largest local communities in the Chifeng area were much greater than those of the West Liao River valley, and the Chifeng population was much more oriented toward larger settlements. It has been revealed that multiple LXJD settlement clusters of roughly contemporaneous periods occupied northeast China [2, 5, 8]. As these clusters consisted of closely packed, variously sized sites in their respective regions, it is suggested by many that they were likely representing “chiefly” polities [2, 23]. Shelach-Lavi proposes that LXJD settlements exhibit relatively high levels of regional integration and there seem to be two hierarchical tiers in terms of site sizes across the LXJD distribution area [2].

## 4. Settlement construction and relative chronology

From the mid-1990s onward, the increase of regional archaeological surveys in northeast China revealed valuable new data that made it possible to research the LXJD settlement patterns, site organizations, and sociopolitical context. However, the revealed differences between various LXJD settlements by archaeological surveys could not indicate whether they were purely spatial divergence or chronological contrast. One huge advantage of archaeological excavations over regional surveys is the opportunity to detect site formation processes and diachronic patterns in settlement development. A wealth of excavated data has been accumulated since the 1970s (Tables 1 and 2), which is exceptionally useful to extract detailed chronometric information for a better understanding of the LXJD settlements. It is worth noting that these LXJD sites were selected for excavation largely because they were among the largest and most impressive examples in the field. The study of LXJD settlements would certainly benefit from a thorough understanding of the landscape in which the community’s most visible sites were situated. A landscape viewpoint enables us to organize settlement data according to two structuring elements: the space, and the method by which the site was built. Following this line, and based on existing archaeological excavations, two architecturally different groups of large settlements can be recognized in the LXJD area (Fig 1): Type A (Table 1), the low-platform sites built with rammed earth/mudbricks and ditches, and Type B (Table 2), stone-constructed high hill forts register a significant canyon-margin location.

Type A sites are those distributed near the valley bottom on low tablelands, with convenient access to water resources. They were built with mudbrick/rammed earth technology and are especially rich in cultural accumulations with dense housing constructions. The average size of known Type A sites is 17,190m^2^ and most sites are more than 10,000m^2^.The Erdaojingzi site in western Liaoning, with a size of 50,000m^2^, is the largest one currently reported. In Type A settlements, multiple tiers of housing remain can usually be observed, overlaying one another from the very bottom to the current floor surface. The houses in Type A sites were largely round or square, either built as semi-subterranean pit houses or ground-level dwellings. A preponderance of arable land is found surrounding large Type A locations in western Liaoning. For example, the Dongshanzui, Fengxia, and Shuiquan sites are reported to be built on secondary terraces [12–14]. These sites are all located less than 50 meters from waterways and agricultural lands, suggesting that dry-land farming very likely formed the most important component of the subsistence economy of these LXJD communities.

Traces of intensive repairs on indoor floors have been identified at many Type A settlements. When a house was first built, the floors were usually paved carefully with a mixture of mud and straw before a layer of lime powder was applied for white and smooth ground. Sometime later, people would clear and level the floor. These ground-leveling activities repeatedly happened throughout the use of Type A settlements. At house F5 of the Fengxia site, for example, six layers of new floors were laid down on top of the former ones, and the bottom layer is the original living surface when the house was first constructed. The total thickness of floor layering found at another Fengxia house, numbered F2, is nearly two meters [12]. Similarly, at the Kalaqin Hedong site, five houses were found overlaying one another and the entire housing foundation is 2.4 meters in depth [27]. Observing the cross-section, archaeologists note that when the sidewall of a house collapsed, new earth and mudbricks were brought in for building new walls and floors, which would be superimposed on the leveled ruins of the old ones. The fact that new walls were built, and houses were reconstructed in their original location, suggests that the residents lived in these Type A settlements for a long period.

In comparison, Type B sites feature well-preserved stone houses, installations, enclosures, and paved roads surrounded by defensive stone walls. Covering an area ranging from 5,000 to 150,000 square meters, the average size of Type B sites is 34,333 m^2^, which is twice as large as that of Type A settlements (17,190 m^2^). Stone-constructed Type B sites are much less rich in anthropogenic deposits compared with Type A sites but showed a stronger sense of fortification. They register a significant canyon-margin location, overlooking major rivers and high on the slopes. Before the construction of massive stone walls, the location of each Type B site was carefully chosen to maximize the effect of natural protection. The sites were then enclosed by stone walls at places where natural cliffs were unavailable, suggesting a great sense in using topography as part of the site protection.

Apart from stone walls, the defensive structures seen in Type B locations were constructed over time. For instance, at Kangjiatun, a stone enclosure was enlarged with bigger walls on the east, west, and south sides in the second phase of the site occupation [17]. It was also during Phase II of the Kangjiatun site that a three-meter-deep moat was constructed outside the stone walls.

External walls have been found in all reported Type B settlements. At the twin-settlement of the Sanzuodian site, both the large and the small settlements were enclosed to the fullest extent, and the widest part of the Sanzuodian stone walls can reach three meters [37]. Facilities with a strong sense of defense have also been found added onto the outer surface of the original stone enclosure as a sort of reinforcement in Type B sites. Known as bastions, or *mamian* in Chinese sources, the circular-shaped watchtower is a type of outer shell structure outside the existing wall body. They were constructed with a pounded-earth core attached to the main walls and a stone semi-circular structure facing outwards. The average size of Sanzuodian and Kangjiatun bastions can reach 5m by 5m, regularly spaced at intervals of no more than seven meters [17, 36, 37]. A great advantage of these circular-shaped towers is that they can provide extra attacking space for the defenders on the walls. Along the 200-meter-long stone walls of the Sanzuodian large settlement, 15 such circular-shaped towers can be identified, and 10 towers have been found attached to the external side of the small settlement [37]. The surviving wall and towers can be as high as four meters, constituting quite an impressive landscape on top of the loess platform.

The locations of these 27 published LXJD sites are marked with color-coded information about their surrounding landscape and constructive methods on a site distribution map (Fig 1), which shows a clear division that Type A sites built on low platforms were more likely dispersed in the Liao River area—the eastern side of the LXJD cultural area, while Type B stone-constructed settlements show a very high concentration on the edge of the Mongolia Plateau. That is to say, the division of LXJD settlements into two major groups suggests both architectural differences and environmental preferences among the LXJD community.

This spatial difference has been touched upon in past literature, in which scholarly interpretations are often linking different construction methods and materials to regional variations. For instance, Bak [41] summarizes in his 2020 Ph.D. dissertation that stone-walled LXJD settlements are concentrated and densely packed east of the Nulu’erhu Mountian, where earthen sites were rarely found. He also noted that the largest settlements and high-density site distribution are visible in the Chifeng-Aohan region. Similar statement can also be found in Xu’s article [42].

In the current paper, we reckon that the LXJD settlement differences in its east and west distributional areas were beyond regional differences and they are crucial to understand the changing pattern of the LXJD society. However, there is a need to note that the currently available data has several limitations. Firstly, the earthen sites constructed in western Liaoning would not have survived as well as the stone settlements, and they were much more prone to erosion during the subsequent human occupation and agricultural activities. Secondly, due to the uneven surface visibility of stone and earthen settlements in the fields, archaeologists and surveyors are not likely to have reported uniformly their findings on the rim of the Mongolian Plateau and western Liaoning. It should be noted that although settlements are recorded to be more nucleated and densely packed in the Chifeng-Aohan area, this does not guarantee that the Chifeng-Aohan region was the only community center of LXJD activities. In other words, the settlement distributional pattern detected from regional surveys, both in terms of settlement core and site sizes, is partly a product of surface preservation and thus has potential archaeological bias. Thirdly, this paper also wants to address that not all LXJD sites were built with fortifications. The above description of characteristics of Type A and Type B sites may convey an impression that fortified settlements were the norm of LXJD society or they were the only type of site form in northeast China. However, in past survey projects, a large number of unfortified and lightly enclosed small sites have been recorded and constitute the greatest number of LXJD settlements: in the survey along the Yin River west of Chifeng, 19 out of 70 identified LXJD sites are undefended [2] and they contain only two or three houses; 130 out of 141 LXJD locations have been found without stone fortifications in the archaeological survey along the middle reach of Banzhijian River, west of Chifeng city [43]. Most of these undefended locations revealed only anthropogenic deposits of the LXJD period, while some contain stone enclosed houses with simple and narrow walls. In contrast with large and central fortified sites, they are identified with much thinner deposits and are located further awar from major water channels and river valleys [44].

Large central fortified settlements, with more houses, storage facilities, handicraft production, and public structures can be regarded as the residential base of high archaeological visibility in the LXJD residential system. In large excavated Type A and Type B sites, excavators reported greater varieties of constructive features, which were not seen in small undefended sites. Structures such as well-constructed bastions and wall enclosures, storage facilities, and large open spaces are found only in large settlements, indicating that they had a different function from the smaller undefended locations and might act as centers of defense, religion, and economic resources [45]. By comparison with large fortified sites, the smaller locations were more likely built as temporary operational and seasonal residential areas for small task groups. Without any report of storage pits or evidence for long-term use, there is only sparse occupational debris, suggesting either that they were not used for long periods or they were only sporadically occupied for specific purposes. On top of these, evidence of maintenance has been reported from various large Type A and Type B sites but has never been recorded in small unfortified locations. All these lines are indicating that the larger settlements were under continuous occupation while the small unfortified sites were intended to be used only for short-term residency for small task groups.

Archaeologically, we have excluded the possibility that Type A and Type B sites were built and used by different cultural groups on the basis that a) the ceramics and stone tools found from all these 27 sites were consistent in their typology, shape, and decorative patterns, indicating that the sites were occupied by population with the same cultural tradition; b) the general inner structures of the residential facility remained the same across both Type A and Type B sites: houses were built in a circular shape and usually equipped with a hearth; outside the main house were storage pits and associated side-houses, which were all enclosed by a wall, forming a courtyard-like residential unit; c) archaeobotanical records recovered from both Type A and Type B sites (Erdaojingzi, Chengzishan, and Sanzuodian) demonstrate a consistent dietary pattern that millet was the principal cereal during both Type A and Type B site occupations [46–48]. All three strands of evidence suggest that during the LXJD period, residents of Type A and Type B settlements used the same set of cooking and serving ceramics, and stone tools, constructed and lived in similarly structured dwelling sites and both depended on dry-land millet farming as the primary subsistence, and they should be regarded as one cultural group.

From the point of field archaeology, stratigraphically based chronology suggests that the stone-constructed LXJD sites along the edge of the Mongolia Plateau (Type B) were built later than the earthen structures (Type A). At the Erdaojingzi site, one of the most well excavated Type A settlements, archaeologists found that in the last periods of its occupation, stones started to be used for constructing walls and as a sort of repair and reinforcing material [19]. Related chronological information has also been revealed from the Kangjiatun site and a recently reported site near Chifeng: Gaojiataizi, where excavators noted that ground-level stone constructed houses were built on top of semi-subterranean pit houses, which were made with mudbrick walls [49, 50]. The different construction materials and the chronological difference were archaeologically very meaningful.

The above evidence points to a suggestion that there was a probable chronological sequence between the LXJD settlements that were constructed with mudbrick/rammed earth techniques in western Liaoning and the ones built in stones on the rim of the Mongolian Plateau. However, as discussed above, ceramics of Type A and Type B settlements did not provide sufficient analytical support to construct a chronological scheme that helps us to understand which settlement type was relatively earlier. Apart from ceramics, it is also unclear from other archaeological remains whether this earth/stone chronological difference was universal in the LXJD area. To test this hypothesis, we bring in all published ^14^C radiocarbon dates and their sample locations for a reassessment of the LXJD chronology.

## 5. Samples

A total of 44 radiocarbon dates [5, 51–55] recovered from 10 sites out of the 27 published locations are listed in S1 Table. They are calibrated with OxCal 4.4 program using the IntCal20 Northern Hemisphere radiocarbon calibration curve [56, 57]. The primary calibration results of the model suggest that the agreement index of one date from Dashanqian (Lab number: ZK-2934) is substantially lower, indicating the sample might be contaminated and thus the data should be removed from the calibration model. The remaining 43 radiocarbon dates that are consistent with the model are taken as indicative of the LXJD culture period.

Following the previous discussion on the landscape, construction materials, and methods, we note that the 43 samples embedded in our calibration model include seven Type A sites: Zhizhushan, Fengxia, Shuiquan, Redianchang, Fanzhangzi, Dadianzi, and Dashanqian; and three Type B sites: Dianjiangtai, Chifeng Location 342, and Sanzuodian. It is worthy to point out that, the Zhizhushan site has yielded both early-period earthen semi-subterrane structures and the late stone-built houses [32]. Combining the site’s original archaeological report with the radiocarbon sample source, we note that the only sample for Zhizhushan comes from pit H42, the earliest stratigraphic layer of the LXJD deposit. This suggests that the Zhizhushan date (ZK-0176) represents the early phase of the site, that is, the period of earthen houses. Therefore, in the following analysis, the date information of Zhizhushan is regarded as part of the Type A occupation.

In the scope of this study, we acknowledge that the small sample size of 27 published LXJD settlements may cloud our vision of the whole occupational period of various LXJD culture. Given that only three stone sites are producing reliable radiocarbon dates, it cannot be overstated that it is so small of a sample size for a comprehensive discussion for the Type B sites chronological sequence. Considering that the number for Type A sites are more than two times the number for Type B locations, the emphasized statistical differences for Type A sites are statistically more amplified than those for Type B sites. In the context of this study, the most significant statistical limitation concerned in this paper relates to two aspects, a. Type B’s small sample size may increase the margin of error for Type B sites occupation period and b. prevent our reading of the relative sequential contrasts between Type A and Type B locations.

## 6. Calibration and results

In the first place, a model is constructed for the whole Lower Xiajiadian occupation period based on the likelihood distribution for each calibrated radiocarbon date. The results are summarized in S1 Table. The modeling results show that the upper boundary 2101–1971 BC (68.3% confidence) gives the date range of the earliest LXJD occupation in northeast China. The lower boundary 1375–1295 BC (68.3% confidence) indicates the period of abandonment of the LXJD settlements.

On top of this, we treat each Lower Xiajiadian site as a whole by using a single-phase calibration model to include samples from the same archaeological site with beginning and ending boundaries to estimate the date range for each site. Within each Phase, the ^14^C data are included using the “R_Date” function. “Sigma Boundary” functions have also been set at the beginning and the end of each sequence, which allows the date range of each sample to spill beyond the dates of the boundaries themselves. Each Phase (the group of the date for one LXJD site) is treated independently so that it is straightforward to reveal if there is any overlapping between the date ranges of any two LXJD sites. According to the original excavation report, none of the 43 radiocarbon dates was collected systematically from the bottom layer contiguously up to the latest layer of a site’s stratigraphy. Therefore, we cannot assume that the date range of each single phase represents the entire occupation period of an archaeological site.

**Fig 2:**
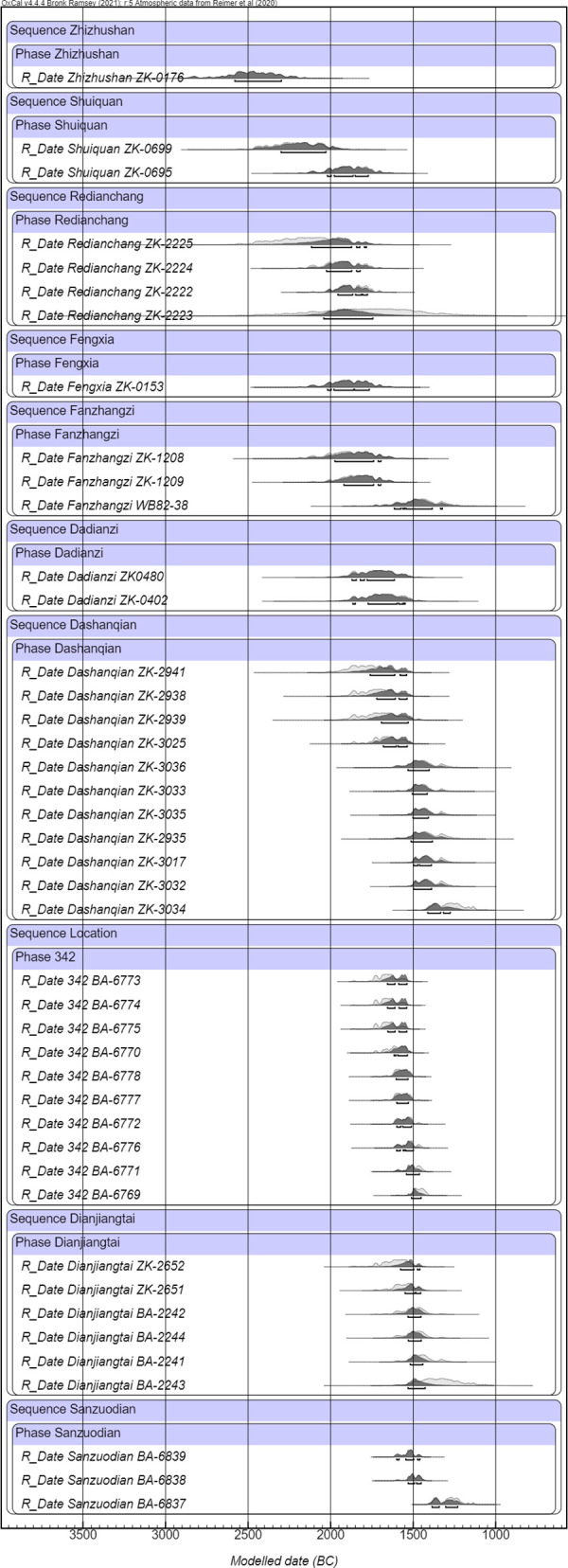
Single-phase calibration for the ten published Lower Xiajiadian sites.

Single-site calibration results shown in Fig 2 suggest Zhizhushan and Shuiquan were among the earliest Lower Xiajiadian sites in northeast China. Later than Zhizhushan and Shuiquan are three Type A locations with considerable overlapping: Redianchang, Fengxia, and Dadianzi. The Dashanqian site is dated from 1661 to 1352 BC (68.3% confidence), which has 11 samples and thus is likely to determine a longer date range. Apart from Dashanqian, most other samples drawn from Type A LXJD locations produced a date range of no later than 1500 BC, representing the early to middle occupation period of the LXJD culture in northeast China.

However, interestingly, it was since ca. 1600–1500 BC that radiocarbon dates for sites, for example, Location 342, in the Chifeng region began to appear, indicating that part of the LXJD population started to move westward onto the edge of the Mongolia Plateau. Fig 2 also demonstrates that there was a long overlapping period between Dashanqian and the earliest dated Type B locations in our data group, Location 342, which the Bayesian model has confirmed to be from 1624 to 1499 BC (68.3% confidence).

Knowing the relative order and archaeological background of Type A and Type B LXJD sites, we then regard them as two contiguous events. Using Bayesian mathematics as a tool for combining radiocarbon dating results with findings from one archaeological context, in this sense, Type A and Type B respectively, we can arrive at a more precise and accurate date range for these different LXJD settlements. To operationalize this combination, the dates of Type A and Type B are presented in OxCal as two “Phases” within a collective “Lower Xiajiadian Sequence”, that the Type A Phase is constrained to lie earlier than the dates of Type B sites. Within each Phase, all the ^14^C data were included by using the “R_Date” function. In the meantime, the “Summary Date” function has also been applied to provide summary statistics for the time ranges of Type A and Type B occupations in northeast China.

**Fig 3:**
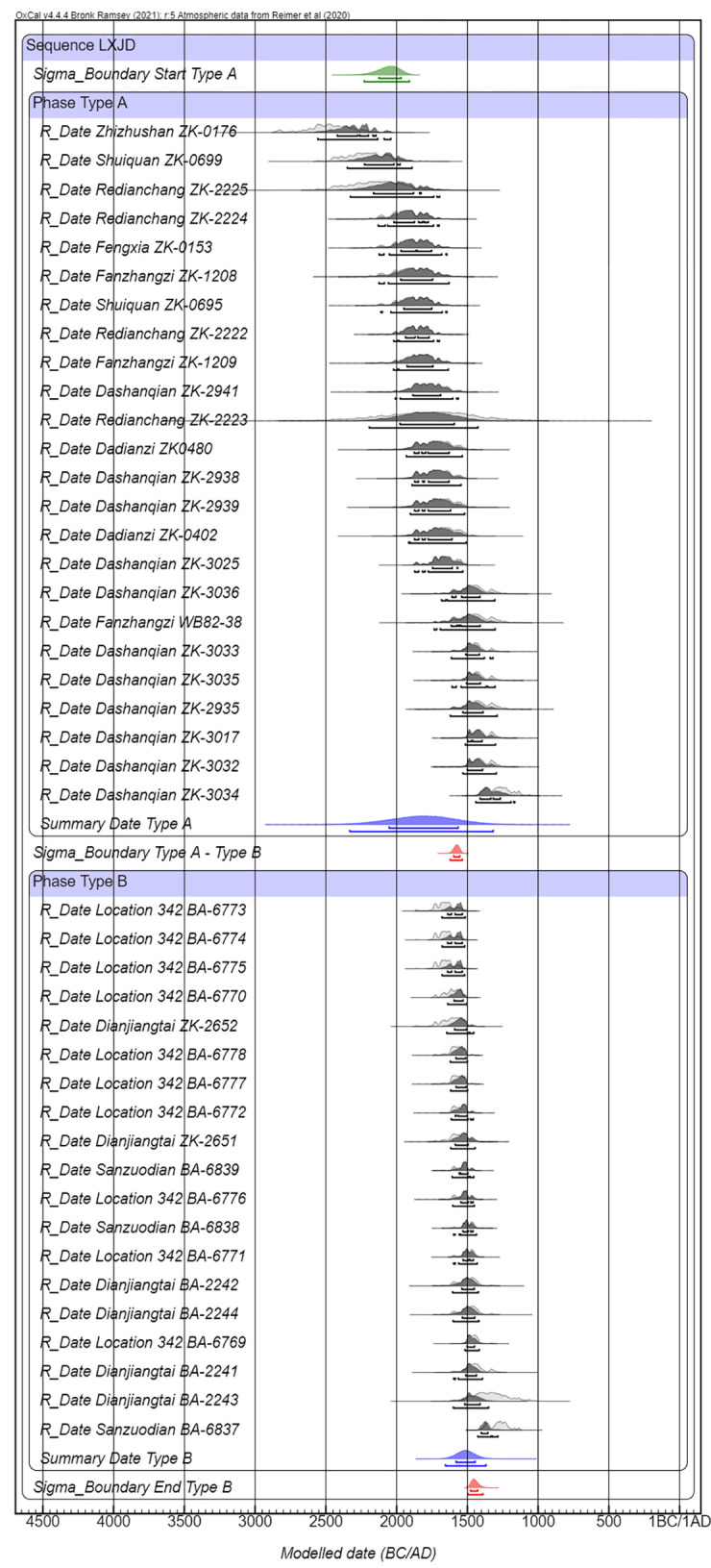
Radiocarbon dates for type A and type B locations.

**Fig 4:**
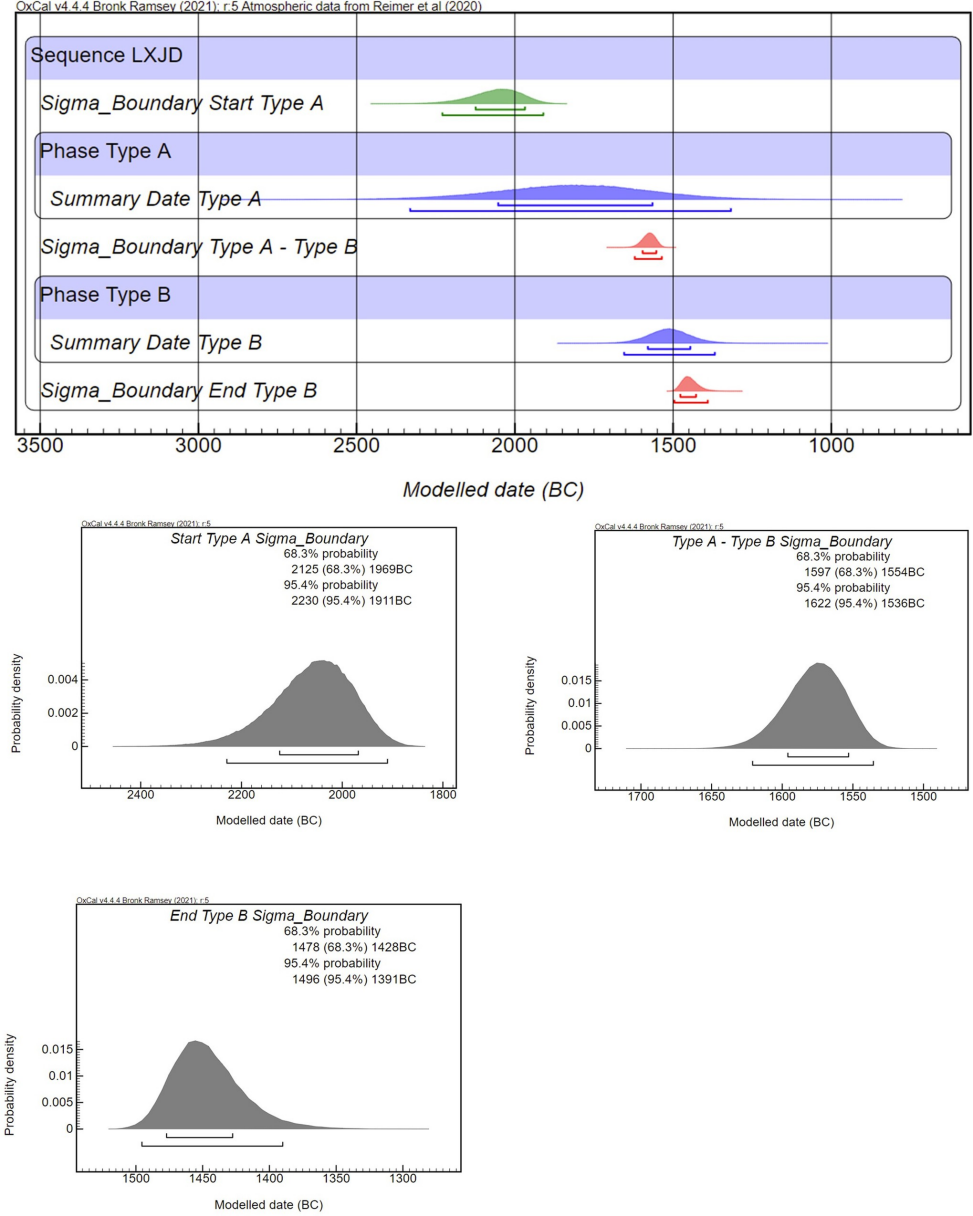
Summary dates and modeled start and end dates for type A and type B sites.

Presented in Fig 3 are the primary calibration results for Type A and Type B sequences. It suggests that the dates for Type A sites range from 2059–1567 BC (68.3% confidence) and Type B locations lasted from 1580–1448 BC (68.3% confidence). More information can be drawn from Fig 4, which indicates the probability distribution and modeled start and end dates for the use of Type A and Type B sites. Taken together, firstly, we understand more precisely the relative order of LXJD site use, that it was during the 16^th^ century BC, 1597–1554 BC (68.3% confidence), and 1622–1536 BC (95.4% confidence), that Type A earthen sites were gradually abandoned, and Type B stone locations were constructed. Secondly, the model with constraints also facilitates the visualization of both types of LXJD sites and their occupational periods. It is visible based on the Bayesian modeling that Type A sites were populated for a longer span than Type B locations (Fig 4). The end date range of Type A highly overlaps with the start date range of Type B sites, indicating that both types of settlements were used in the mid-second millennium BC. This also underscores the possibility that the remains of stones discovered from the upper deposition layers at several of the Type A settlements were constructed contemporarily with the stone episode seen in the Chifeng region.

## 7. Discussion

### 7.1 Chronological implications

The division of Lower Xiajiadian settlements into two architectural groups has been shown to have both constructive and chronological parameters. The revealed radiocarbon chronology for Type A and Type B sites have affirmed our first hypothesis that Type A earthen sites in western Liaoning were the earliest locations being constructed and occupied, followed by stone settlements on the edge of the Mongolian Plateau. A more crucial fact is that it took less than 100 years, or potentially 50 years (1596–1554 BC with 68.3% confidence, and 1621–1536 BC with 95.4% confidence) for the LXJD people to shift their residential centers onto the Mongolian highlands, where they adopted stone as a kind of new and primary material to construct and fortify their residential houses. Considering that the whole date range of LXJD activity in northeast China is around 700 years, the decision made by the residents to change their settlements must have happened within a brief period, and possibly out of necessity in a dire circumstance.

Till around 1600 BC, settlements in western Liaoning were still in use, probably until the late 16^th^ century BC. In the meanwhile, some settlements were being repaired with stone in their late period of occupation, while other sites were abandoned, and the residents move gradually into southeastern Inner Mongolia. We argue that this was the reason why radiocarbon dates overlapped between sites in western Liaoning (e.g., Dashanqian) and Chifeng (e.g., Location 342) from around 1600 to 1400 BC. The evidence of stone being used as repair or reinforcement material in several Type A settlements may take place contemporaneously with the later stone episode seen in the Chifeng region. However, without further radiocarbon dates on materials from both late Type A and early Type B locations, it is difficult to say how long the period was between large-scale stone fortifications in the Chifeng region and the early stone layers found at some of the Type A sites. Undisputedly, it was after 1500 BC that stone gained ultimate importance in the LXJD culture and large-scale stone-fortified sites, best represented by Sanzuodian and Kangjiatun, were built and used until around 14^th^ century BC.

The contrasts between Type A and Type B locations cannot be overstated as the growing importance of fortification was embedded in the planning, building, and constructive materials of all excavated Type B stone settlements. Besides the heavy use of stone, three other differences between Type A and Type B are settlement size, topography, and cultural deposit. The size of average Type B sites (34,333 m^2^) is nearly twice as large as that of typical Type A settlements (17,190 m^2^), indicating that Type B sites were intended for residential purposes for a more gathered population and more diverse communal functions. Type B high hillforts were naturally enclosed by their locations on valley margin areas with steep slopes. They were further protected by stone walls at places where natural cliffs were unavailable, and this characteristic has been illustrated at multiple sites such as Xindian [8], Shangjifangyingzi [18, 20], Kangjiatun [17], and Sanzuodian [36, 37]. Using topography as part of the site protection has not been reported from any known Type A sites, and this surely suggests that Type B residents were experiencing a much more hostile environment than those living in Type A locations. Furthermore, the anthropogenic deposits left by LXJD people in Type B stone sites are much fewer than those in the Type A settlements, plus there is no evidence of repairment or reinforcement of existing architectures has been noted in Type B settlements. From all three perspectives, these remarkable contrasts are highly indicative of social changes that took place in the LXJD area in the mid-second millennium BC, which invite multiple explanations.

The contrast between Type A and Type B sample sizes have been noted in the preceding text, and we acknowledge that due to the small sample size for Type B locations, the observed pattern shown and discussed above may not represent the chronological pattern of archaeological reality. The most possible disadvantage Type B’s small sample size brings is an uncoverage bias and a lack of data response, which suggests that some Type B sites do not have the opportunity to be included in our radiocarbon calibration and interpretation. In particular, the small sample size for Type B sites affects the reliability of our discussion on the overall time period for Type B locations, which was likely more extended than already observed and their start and end dates would be more accurately presented as more samples become available in the future. Restricted by data availability, the current recognized chronological order may also be misleading in that the calculated coeval period of Type A and Type B sites lacks sound statistic support, and it is greatly hoped that future research and lab works will substantially expand the radiocarbon dating dataset of more LXJD stratigraphic layers and across multiple sites of both Type A and Type B.

### 7.2 Chronology and LXJD’s millet farming

It has been discussed by many scholars that northeastern China was a significant center where millet agriculture first started [58–60]. Archaeobotanical records of the LXJD culture sites indicate that local subsistence was based on dry-land millet farming, supplemented with animal husbandry [5, 20, 33, 46–48, 59, 61–63].

Sun et al. [48] reported that 181,685 carbonized foxtail millet and 41, 266 broomcorn millet seeds have been identified from the 435 L of soil in 77 samples at the Erdaojingzi site (Type A). Both types of millets, together with a small number of soybeans and cannabis, accounted for 89.10% of the total plant remains. A total of 314 carbonized foxtail millet seeds and 98 carbonized broomcorn millet seeds were identified from 100 samples at the Chengzishan site (Type B) in Lingyuan County, Chaoyang, Liaoning Province [46]. However, an archaeobotanical study of the 103 soil samples from Type B site Sanzuodian yields a contrasting result from the Type A settlements: 9,665 carbonized foxtail millet seeds and 99,348 carbonized broomcorn millet seeds were identified, accounting for more than 99% of the total floated plant remains [47].

It has been demonstrated by environmental archaeological studies that a warm and humid climate might have promoted the early expansion of the LXJD community in northeastern China [5, 64]. Carbonized wood remains from western Liaoning and two Type A sites (Dashanqian and Dadianzi) all suggest a primary vegetation and weather system of a noticeably higher temperature and annual rainfall than present-day [65, 66]. An 8.5m-long sediment core has been extracted from Dali Lake, on the eastern edge of the Mongolian Plateau. The core was analyzed at 1-cm intervals for total organic and inorganic carbon concentrations, which shows that the water level of Dali Lake reached its highest stand from 7,200 to 4,600 cal. yr BC. The lake level then exhibits high-frequency, high-amplitude fluctuations after 4,600 cal. BC. From ca. 1,450 cal. BC, the lake level has displayed a notable declining trend and fell to the lowest stand at three intervals of ca. 1,150–650 BC, AD 350–850, and AD 1450–1800. The high lake levels during the early to middle Holocene, according to the author, marked the influence of the East Asian summer monsoon over the lake region when the monsoonal rainfall belt pushed to its northern limit. Conversely, the lower and highly variable lake levels after 4,600 BC, especially after ca. 1,450 BC, suggests that the monsoonal precipitation was reduced, and the summer monsoon was dramatically weakened in the late Holocene. A few other climatic reconstructions have equally characterized northeastern China as subject to decreasing precipitation after the mid-second millennium BC [67]. That is to say, a favorable climate promoted agricultural activities during the early phase of the Lower Xiajiadian culture, followed by a noticeable environmental deterioration, in the form of climate instability with periodic drought events after the mid-second millennium BC.

Given the chronological information and climatic background concerning Type A and Type B settlements, it could be speculated that the proportion of broomcorn millet grew dramatically from the early to the late period of the LXJD occupation as the environment deteriorated. Broomcorn millet, with relatively stronger drought and saline-alkaline resistance than foxtail millet, was popular outside central China [68, 69]. It was a millet variant that was more often associated with farming groups in northern China and pastoralists of the Eurasia steppe, who lived in the drier environment and cannot afford high-investment agriculture. The overwhelmingly high proportion of broomcorn in Sanzuodian’s archaeobotanical profile may be one of several attendant consequences of LXJD’s settlement relocation. After 1500 BC, possible resource scarcity intensified due to climatic deterioration in northeast China. With decreasing precipitation and climate instability, the LXJD community living in Type B stone settlements probably had given up foxtail millet, shifting their priority to the more drought-tolerant broomcorn millet.

In this context, the subsistence pressure may be alleviated both by an expansion away from the earlier core region and a shift of subsistence economy from foxtail millets to more drought-resistant broomcorn millets, and therefore, this is the period we see in the radiocarbon profile when a few earthen sites were dated contemporary to stone settlements in the Chifeng region. From the data now available, archaeologists have not observed a clear change in the major crop in west Liaoning, but this does not exclude the possibility that a shift from foxtail to broomcorn millets also took place in this region. Further evidence and research are needed to explore how the interplay between environmental dynamics, settlement distribution, and the change in crop regime. But what this study address is the importance to establish a chronological point of view, through which a comprehensive reading of climate instability and human behavior change can be achieved.

### 7.3 Chronological difference and the theory of intra-community conflict

As has been noted above, the recognition of contrasts in settlement patterns between the Chifeng area and western Liaoning has been interpreted as a regional difference, instead of chronological variance. The sociopolitical context within which the regional LXJD settlement clusters worked and functioned has been extensively explored through the perspective of resource and conflict [22, 70], and the observed settlement variabilities are mostly understood as reflecting different degrees of competition and intra-community conflict between the east and west areas of the LXJD culture. In areas of less population density, for instance, the Upper Daling River area, less resource pressure would lead to less inter-community competition and less opportunity for elite advancement and power centralization. In contrast, in areas of higher population levels, there was correspondingly higher pressure on subsistence resources and consequently, a higher level of internal conflict, even warfare, between different LXJD communities [22]. Under this circumstance, the LXJD communities living in the Chifeng region competed militarily with each other for access to subsistence resources, which drove demographic growth as communities with larger populations would have a competitive advantage in the form of abundant labor, larger military troops, and more elaborate fortifications [22]. The impressive stone walls seen at the Sanzuodian site have also been interpreted as a visual symbol of communal integration, advanced social mechanisms, as well as the status competition of the local elites [70]. Likewise, Shelach-Lavi thinks the key position of large elaborate settlement structures appeared in most parts of north and central China during this period, either seen as representative of elite residences, or buildings used for public functions, all reflected the social control and the legitimization of political power of the local leaders [71].

Apart from the more visible stone fortifications, a full-coverage regional survey in the Chifeng area has also disclosed a large number of isolated, hilltop LXJD sites. However, an intensive survey in the Upper Daling region recovered only one unmistakable small hilltop site, at the center of the northwesternmost site cluster in the survey area [21]. The survey data suggests that stone sites smaller than two and a half hectares of the Chifeng area were arranged in groups and located further away from the main river system [2]. Built with stone and located on the edge of the Mongolia Plateau, these hillside sites have smaller but denser houses than large, fortified settlements, and are surrounded by quite simple and narrow stone walls. Stone enclosures found at these locations are much less labor-intensive in comparison with those at large, central settlements. Archaeologists have also identified much thinner anthropogenic deposits in these small, hilltop sites, normally are a few potsherds inside the houses or near the wall foundations [72]. Without any report of storage pits or evidence for long-term use, there is only sparse occupational debris in these small, isolated hilltop locations, the function of these hilltop sites has been interpreted as a temporary shelter from raiders/military threats under the Resource and Conflict scenario [22].

In this paper, the chronological implications offered by reanalyzing all available radiocarbon data of excavated LXJD sites suggest that more explanations other than Resource and Conflict are needed to account for the change in settlement construction and the increasing elaboration of LXJD fortifications in the Chifeng region. We suggest that the differences revealed in LXJD settlements’ landscape and construction should be considered not only spatially, but also chronologically.

Predicated upon the Resource and Conflict scenario, one could certainly argue that the transition from Type A earthen settlements in low-platform locations to Type B stone fortified hill-side sites over time is a pure autochthonous societal change due to resource scarcity. However, as has already been acknowledged by Drennan, et al., it is not possible to make a meaningful estimate of the carrying capacity of either the Chifeng or the Upper Daling River area [22], because current archaeological data do not provide demonstrable detailed information on local subsistence resources, such as soil fertility, water, animal, habitat, and weather stability. Considering the much higher population levels of LXJD culture than in any previous periods, the pressure on subsistence resources is surely one possibility in the discussion on the change of LXJD settlements, but whether or not these resource stresses have grown to the point that inter-community hostilities became widespread that military conflicts were so universal, are difficult to demonstrate.

In addition, one often overlooked issue in existing scholarship on the Resource and Conflict model is the lack of archaeological evidence of violence during the LXJD period. Neither has any data on trauma or individual killed by violence conflicts been revealed by full-coverage regional surveys and excavations of LXJD sites. Therefore, we could not say for sure at this stage that the LXJD settlement clusters built with defensive structures were used for intra-community military confrontation. More supporting archaeological data would be helpful to supplement and underpin the existing Intra-polity Conflict hypothesis.

### 7.4 Possible external pressure and threat

One underappreciated issue in the above-mentioned regional archaeological surveys is LXJD’s association with adjacent cultural entities. Social trajectories of northeast China during this period were far from entirely independent of external influences, and there is good reason to look for outside pressure that also contributed to LXJD’s settlement variability. Recent genetic studies of human samples from the Dadianzi cemetery and Erdaojingzi site indicate that there was substantial genetic admixture from the Central Plain during the LXJD period [73, 74]. Late Neolithic genomes associated with the LXJD culture have been revealed to overlap greatly with the ancient Yellow River cluster in Central China, suggesting a strong northward influx from a Yellow River-related population between the Middle and Late Neolithic periods [75]. The genetic admixture itself in nature would not suggest whether the influx of southern populations was hostile or amiable, but in this context, it is worth pointing out that the change of LXJD residential centers and construction materials and methods cannot be neglected, or taken only from an internal perspective. Instead, it would benefit from a discussion of external impacts and contemporary communities.

**Fig 5:**
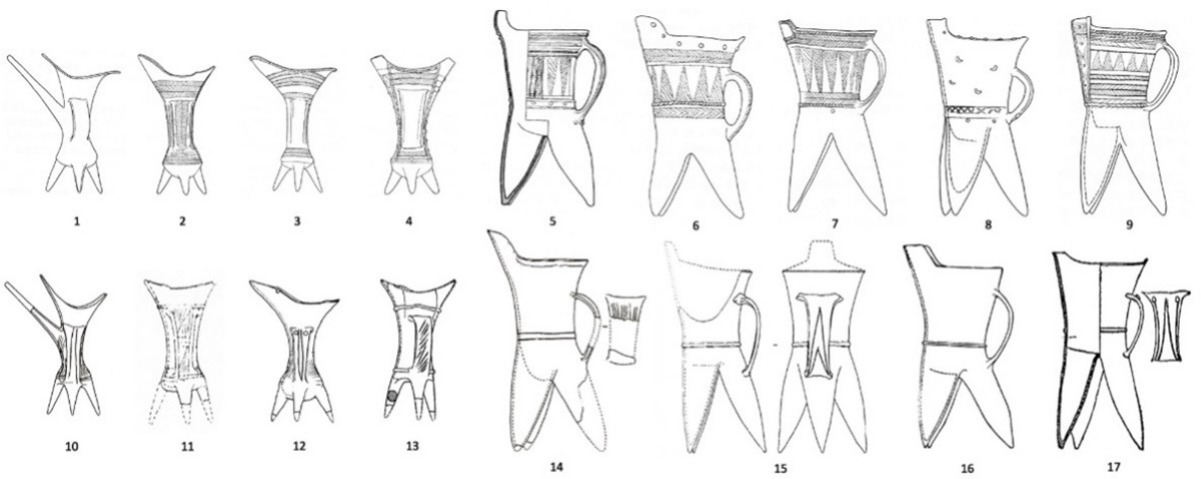
Comparison of pottery tripod vessels from Dadianzi (1–9) and Erlitou (10–17).

Clear traces of such far-flung connections are more exhibited in the burials at Dadianzi. At Dadianzi, 24 unmistakable Erlitou elites’ drinking/pouring ceramics (11 gui 鬶, 12 jue 爵, and 1 he 盉) were deposited in 13 elaborate tombs [33] (Fig 5). Their close resemblance to their southern counterparts from Central China almost certainly suggests a distinctive influence from areas outside of northeast China.

After 2000, the Chinese academics were involved in a great number of discussions on the nature of LXJD’s stone fortification and their social functions. Many of the scholarly works emphasize the strong military/defensive qualities of the stone-constructed settlements [76–79]. The distribution and clustering of stone settlements around the Chifeng region remind many scholars of the famous Great Wall, later constructed along the northern edge of Central Plains. Han Jianye [80], especially, argues for a narrative that the northern border had constantly been an area of conflict between the southern agricultural communities and mobile pastoral groups further north. Referencing climate and ecological data within this monsoon marginal area, Han thinks that these south-north conflicts have largely resulted from economic distinctions and an imbalance of regional subsistence resources. Han’s perspective addresses an important ecological background in which populations with different subsistence strategies became hostile to each other during the second millennium BC. In this scenario, the change in LXJD’s settlement construction can be attributed to increasing pressure from the pastoralist groups of the Mongolian Highland.

By comparing the date range of the LXJD culture with contemporary archaeological cultures in the Chinese Central Plains, we note that external pressure from south of the LXJD area was also a possibility that might have triggered a settlement change from earthen structures to stone fortifications. Considering the date range of LXJD culture, the Liao River valley was long populated before the rise of the Erlitou complex (ca.1750-1500 BC), the earliest bronze-casting culture in Central China. The LXJD group remained active in northeast China beyond the end of Erlitou in ca. 1500 BC. However, Type B highland stone locations appeared in large quantities soon after the fall of Erlitou, a period contemporary with the early phase of the Shang regime (ca. 1500–1045 BC), the Erligang phase (ca. 1500–1300 BC). Archaeological research in past decades has offered abundant data indicating that Erligang was a period featuring territory expansion. The extent of Erligang Shang power has been mapped based on ceramics, bone objects, jade items, weaponry, and ritual bronzes across a large area. The southern, eastern, and western outwards from the core of western Henan have been well demonstrated by the site of Panlongcheng [81] in the Yangtze River valley, Daxingzhuang [82] in the Shandong peninsula and Laoniupo [83] in Shaanxi, respectively. Scholars have raised the model of the Erligang expansion and its military occupation across vast areas, though archaeologists have not been able to identify a clear northern frontier of Shang territory during the Erligang period. Instead, in the Yan Mountain region, the confrontation region between Shang and the LXJD area, a large number of bronze weapons and ceramics of Shang culture have been found in sites such as Liujiahe [84], Dachang Datuotou [85], Zhangjiayun [86–88] and Zhangying [89]. These archaeological data carry arguably Shang cultural elements and are seen as Shang’s presence in the southern rim of the LXJD area [90, 91].

Chronologically, the date at which the LXJD group shifted their residential centers and constructed distinctive stone fortified settlements was concurrent with the period when Shang political influences were pushed into areas outside of Central China and major Erligang territory expansion was observed archaeologically [6, 92, 93]. However, it has been pointed out by Campbell that the thinking of the Central Plains Bronze Age horizon as a homogeneous militaristic expansion into the periphery region, in this case, northeast China, may have oversimplified the situation [94]. Instead, he suggests seeing Zhengzhou as a node in far-reaching and constantly changing networks of warfare, alliance, ritual, trade, tribute, and other things [95]. Although Campbell has listed out there are multiple regional Erligang variants and local cultural traditions (including LXJD), the argument itself could not deny the fact that, during the mid-second millennium BC, Zhengzhou had formed a center of an unprecedentedly large and integrated distribution of sites and metropolitan-type material culture, which showed Central Plains’ coercive political power and potential military ambition.

Taking up the idea of the “prototype Great Wall” further, Zheng Shaozong and Sun Yonggang suggest that the chains of the heavily fortified LXJD stone fortress may be architectural remains of a northern state contemporary with the Central Plains Shang polity [78, 79]. Although there is no specific discussion on the issue of whether or not the LXJD group had been recorded in the historical documents, several Chinese researchers have touched upon this topic and quite a few interesting viewpoints have been raised. For example, Zou Heng supports the view that the LXJD remains is related to the Sushen group [96], while Zhang Zhongpei thinks the LXJD community might be the Yi tribe mentioned in the Shang text [97].

Overall, the change in residential sites as revealed from archaeological data could represent a particular adaption of the LXJD population to a specific situation of external pressure that stimulated a necessary defensive response in order to protect their settlements and territory. In this scenario, this paper argues for new perspectives to view the change of LXJD territory center and residential structures as part of the local group’s efforts in response to the pressure outside of northeast China.

### 7.5 Shared stone constructions along China’s northern frontiers

Since the early to mid-third millennium BC, southern central Inner Mongolia was a region where stone-walled settlements were distributed. During the subsequent late Longshan period (ca.2200-1900 BC), stone sites began to appear in northern Shaanxi and Shanxi provinces, and it seems that areas including the LXJD, were drawn into an ever-growing northern sphere of activity, featuring the construction of large stone-walled settlements.

The concentration of Late Neolithic sites in the Loess Plateau is as astonishing as the LXJD culture in northeast China. More than 2,200 Longshan periods (2600–1900 BC) sites have been found in Shaanxi province, with a great spatial contrast between the north (Ordos region) and the south. There are more than 1400 Longshan sites reported from northern Shaanxi, while only 44 locations have been found in the southern part of the province. A cross-country archaeological survey confirmed that among the 820 Neolithic sites found in Yulin area 榆林地區 (northern Shaanxi), 125 belong to the Yangshao culture and 695 to the Longshan culture period [98]. That is to say, compared with the Yangshao and Miaodigou II periods (ca.4200- 2600 BC), the distribution of archaeological sites during the last centuries of the third millennium BC displayed a tremendous north-ward expansion into the highlands.

Located on the northern edge of the Loess Plateau and bordering the Mu Us desert to the north, the site of Shimao 石峁, featuring large stone fortifications, jades, and early metal artifacts, has been under excavation since 2011 [99]. Several pits containing human skulls excavated under the main structure of the east gate have produced radiocarbon dates between 2300–1800 BC [100, 101].

Visually impressive, Shimao fortifications exhibit many sophisticated and well-designed features that show both similarities and differences with the LXJD stone settlements. Firstly, the stone blocks exploited in Shimao were believed to be quarried from locally available sandstones and had been carefully selected and polished to produce a good consistency [99]. By comparison, the stone walls and external towers found at the LXJD settlements were constructed with unshaped natural stones, thus producing quite uneven surfaces. In the Chifeng area, the lack of surface finishing at large stone-walled sites could considerably limit the investment of labor.

Secondly, at both Shimao and LXJD settlements, semi-circular stone-built external bastions (*mamian* towers) were incorporated into the fortification system. These outward-facing towers could hold several defenders and their weapons to inflict flanking fire on the attackers. However, the stone walls and bastions at Shimao were significantly larger, both in length and volume, than the ones of the LXJD culture.

Thirdly, in thinking about the scale of the Shimao enclosure and the possible ritual function of the Huangcheng Tai terrace and skull pits, it is highly likely that it was a primary center for ritual and ceremonial activities. Although there have been houses and storage facilities reported near the Huangcheng Tai terrace, further archaeological work is needed at Shimao to confirm its inner structures and functions. As for the LXJD sites, we have shown that there were functional variations between large and small, defended and undefended settlements, but on the whole, the stone constructed LXJD sites were more used as residential space and were less ritually significant.

In the Khakass-Minusinsk depression north of Altai, the construction of stone fortifications intensified in the late third millennium BC, indicating increased competition for resources, pastures, people, and trade [102]. It is also from the mid-third millennium BC that networks of stone fortresses first flourished along the northern rim of China’s loess highlands. Featuring extensive stone-built settlements, archaeological remains of the Laohushan 老虎山 culture are distributed north of the Ordos to Daihai region, constituting the southern counterparts to the stone fortifications from Tuva to the Cis-Baikal region north of Altai [103, 104]. In this narrative, the construction of Shimao’s stone-walled enclosures and platforms appeared to have incorporated architectural traditions from south Siberia and may also directly benefit from Laohushan, its immediate predecessors north of the loess highlands. The unusual practice of building similar large-scale stone fortifications was clearly shared among northern communities, and these activities, mostly predating LXJD, may in the very beginning triggered by external incentives.

To summarize, the close proximity both in chronology and construction, between Shimao and LXJD presents us with a shared stone construction entity across northern China. Featuring large-scale stone fortifications, these massive northern sites and large population aggregations have significantly changed our perspectives on the social developments north of the Central Plains before the rise of Erlitou (1750–1530 BC) and the subsequent Shang dynasty (1500–1045 BC). The ability to gather and manage labor on such a large scale indicates that these northern communities had high-level social organizations that were not at all inferior to the Central Plain cultures.

## 8. Conclusion

Through an integrated approach of combining published ^14^C data from different sites with archaeological backgrounds, our analysis suggests that the LXJD settlements with earthen structures were earlier than those constructed with stones. More importantly, the chronological data revealed in this article sheds light on how to understand the adoption of stone settlements and the shift of settlements to high and defensive locations. If placing the events of LXJD settlement transformation against a larger geopolitical background, this paper points out that the occurrence of the residential change in northeast China is by no means accidental. Based on the current ^14^C profile, the transition of LXJD settlements occurred during 1600–1500 BC, which coincided with substantial environmental deterioration, a flourishing of mobile pastoral lifeway in the Eurasian steppe, as well as an expansion of Erligang Shang from the Central Plains. The underlying social and ecological dynamics of LXJD’s settlement relocation and change of site construction material need to be discussed from multiple angles.

The research on radiocarbon data has been regarded as a powerful tool to provide accurate conclusions on the chronology and periodization of archaeological materials. In this article, we have shown that radiocarbon analysis can not only provide archaeologists with chronological information about the sites and artifacts but also prompts researchers to think about related social issues and ask more diverse questions. Restricted by the LXJD radiocarbon dataset, this study’s design, implementation and interpretations of Bayesian analysis on the LXJD settlements, especially Type B sites, have potential statistic limitations. We are looking forward to seeing more scientific excavations and radiocarbon dating works on the LXJD remains and surely open to more robust statistic extrapolations and new archaeological discussions on the LXJD settlement construction and chronology. Further evidence from other archaeological research aspects also needs to be brought in, and we hope the calibrated results and radiocarbon analysis offered here have lent a provoking perspective for future discussion on the Lower Xiajiadian culture.

## Supporting information

S1 Table Radiocarbon dates for the Lower Xiajiadian culture. (DOCX)

## References

[1] Guo DS. Lower Xiajiadian Culture. In: Nelson SM, editor. The archaeology of northeast China: Beyond the Great Wall. London: Routledge; 1995. pp. 147–181.

[2] Shelach-Lavi G. Leadership Strategies, Economic Activity, and Interregional Interaction. Fundamental Issues in Archaeology. New York: Kluwer Academic/Plenum; 1999.

[3] Weneuju Guojia. Zhongguo Wenwu Dituji: Shanxi Fence (Atlas of Chinese Cultural Relics: The Volume of Shanxi). Beijing: Cultural Relics Publishing House; 2003. In Chinese.

[4] Wenwuju Guojia. Zhongguo Wenwu Dituji: Liaoning Fence (Atlas of Chinese Cultural Relics: The Volume of Liaoning). Beijing: Cultural Relics Publishing House); 2009. In Chinese.

[5] Chifeng International Collaborative Archaeological Research Project. Settlement Patterns in the Chifeng Region. Pittsburgh: Center for Comparative Archaeology; 2011.

[6] Liu L, Chen X. The Archaeology of China: From the Late Paleolithic to the Early Bronze Age. Cambridge: Cambridge University Press; 2012.

[7] Zhang C. Longshan-Erlitou— Zhongguo Shiqi Wenhua Geju de Gaibian yu Qingtong Shidai Quanqiuhua de Xingcheng (Longshan-Erlitou Cultures — Changing Cultural Patterns in Prehistoric China and the Emergence of the Globalization in the Bronze Age). Wenwu (Cultural Relics). 2017; 6: 50–59. In Chinese.

[8] Xu GJ. Chifeng Yingjing He, Yin He Liuyu de Shichengzhi (The sites of the stone cities in the Yingjing River and Yin River valleys at Chifeng). In: Zhongguo Kaoguxue Yanjiu Bianweihui (Editorial Board of Chinese Archaeological Research), editor. Zhongguo Kaoguxue Yanjiu: Xianai Xiansheng Kaogu Wushinian Lunwenji (Chinese Archaeological Research: Collected Essays in Commemoration of Xia Nai’s 50 Years of Archaeological Work). Beijing: Cultural Relics Publishing House; 1986. pp.82–100. In Chinese.

[9] Kosaku H, Seiichi M. Chifeng Hongshanhou. In: Kosaku H, Seiichi M, editors. Archaeologia Orientails, Series A, Volume VI. Tokyo: Far Eastern Archaeology Society of Japan; 1938.

[10] Rehesheng Bowuguan Choubeizu. Rehesheng Lingyuanxian Haidaoyingzicun Faxiande Gudai Qingtongqi (The ancient bronzes found at Haidaoyingzi Village, Lingyuan County, Rehe Province). Wenwu Cankao Ziliao (Cultural Relics). 1955; 8: 16–27. In Chinese.

[11] Tong ZC. Chifeng Dongbajia Shichengzhi Kanchaji (The survey of Dongbajia at Chifeng). Kaogu (Archaeology). 1957; 6: 15–22. In Chinese.

[12] Liaoningsheng Wenwu Ganbu Peixunban. Liaoningshen Beipiaoxian Fengxia Yizhi 1972 Nian Chun Fajue Jianban (The site of Fengxia at Beipiao County, Liaoning Province). Kaogu (Archaeology). 1976; 3: 197–210. In Chinese.

[13] Bowuguan Liaoningsheng. Neimenggu Chifengxian Sifendi Dongshanzui Yizhi Shijue Baogao (The excavation of Dongshanzui site at Sifendi, Chifeng County, Inner Mongolia). Kaogu (Archaeology). 1983; 5: 420–429. In Chinese.

[14] Bowuguan Liaoningsheng. Jianping Shuiquan Yizhi Fajue Jianbao (The excavation of Shuiquan site at Jianping). Liaohai Wenwu Xuekan (Journal of Liaohai Cultural Relics). 1986; 2: 11–29. In Chinese.

[15] IA CASS. Neimenggu Kalaqinqi Dashanqian Yizhi 1996 Nian Fajue Jianban (The excavation of Dashanqian site at Karqin Banner, Inner Mongolia, 1996). Kaogu (Archaeology). 1998; 9: 43–49. In Chinese.

[16] IA CASS. Neimenggu Kalaqinqi Dashanqian Yizhi 1998 Nian de Fajue (The excavation of Dashanqian site at Karqin Banner, Inner Mongolia, 1998). Kaogu (Archaeology). 2004; 3: 31–39. In Chinese.

[17] Liaoningsheng Kaogu Yanjiusuo. Liaoning Beipiaoshi Kangjiatun Chengzhi Fajue Jianbao (The excavation of Kangjiatun site at Beipiao, Liaoning). Kaogu (Archaeology). 2001; 8: 31–44. In Chinese.

[18] Neimenggu Wenwu Kaogu Yanjiusuo. 2006 Nian Chifeng Shangjiyingfangzi Shichengzhi Kaogu Fajue Baogao (The excavation of Shangjiyingfangzi site at Chifeng, 2006). Beifang Wenwu (Cultural Relics in Northern China). 2008; 3: 22–26. In Chinese.

[19] Neimenggu Wenwu Kaogu Yanjiusuo. Neimenggu Chifengshi Erdaojingzi Yizhi de Fajue (The excavation of Erdaojingzi site at Chifeng, Inner Mongolia). Kaoghu (Archaeology). 2010; 8: 13–26. In Chinese.

[20] Neimemggu Wenwu Kaogu Yanjiusuo. Chifeng Shangjiyingfangzi yu Xiliang (Shangjiyingfangzi and Xiliang at Chifeng). Beijing: Science Press; 2012. In Chinese.

[21] Peterson CE, Lu XM, Drennan RD, Zhu D. Upper Daling Region Settlement Dataset; 2014. Database: Comparative Archaeology Database, University of Pittsburgh. Available from: http://www.cadb.pitt.edu.

[22] Drennan RD, Peterson CE, Xueming L, Da Z, Shenguang H. Settlement and social dynamics in the upper Daling and Chifeng regions of northeastern China. Asian Archaeology. 2014; 2:50–76.

[23] Chifeng International Collaborative Archaeological Project. Regional Archaeology in Eastern Inner Mongolia: A Methodological Exploration. Beijing: Science Press; 2003. In Chinese.

[24] IA CASS. Beipiao Shi Xidachuan Xiajiadian Xiaceng Wenhua Yizhi (The site of Lower Xiajiadian Culture at Xidachuan in the city of Beipiao). In: Zhongguo Kaogu Xuehui (Society of Chinese Archaeology), editor. Zhongguo Kaoguxue Nianjian 1999 (Yearbook of Archaeology in China 1999). Beijing: China Social Sciences Press; 2001. pp.149–150. In Chinese.

[25] Liaoningsheng Kaogu Yanjiusuo. Chaoyang Luoguodi Xiajiadian Xiaceng Wenhua Yizhi Fajue Baogao (The excavation of the site of Lower Xiajiadian Culture at Luoguodi in Chaoyang). In: Liaoningsheng Kaogu Yanjisuo, editor. Liaoningsheng Daolu Jianshe Kaogu Baogaoji (The collection of archaeological reports in the process of the railway construction in Liaoning Province). Shenyang: Liaoning Nationalities Press; 2004. pp. 95–163. In Chinese.

[26] IA CASS, 1988. Chaoyang Shi Redianchang Xiajiadian Xiaceng Wenhua Yizhi (The site of Lower Xiajiadian Culture at Redianchang in the city of Chaoyang). In: Zhongguo Kaogu Xuehui (Society of Chinese Archaeology), editor. Zhongguo Kaoguxue Nianjian 1987 (Yearbook of Archaeology in China 1987). Beijing: China Social Sciences Press; 1988. pp.125. In Chinese.

[27] Bowuguan Liaoningsheng. Liaoning Jianpingxian Kalaqinhedong Yizhi Shijue Jianbao (The excavation of Kalaqinhedong site at Jianping County, Liaoning Province). Kaogu (Archaeology). 1983; 11: 973–998. In Chinese.

[28] IA CASS. Xingcheng Xian Xianlingsi Xiajiadian Xiaceng Wenhua Yizhi (The site of Lower Xiajiadian Culture at Xinlingsi in the county of Xingcheng). In: Zhongguo Kaogu Xuehui (Society of Chinese Archaeology), editor. Zhongguo Kaoguxue Nianjian 1985 (Yearbook of Archaeology in China 1985). Beijing: China Social Sciences Press; 1985. pp.124–125. In Chinese.

[29] Neimenggu Wenwu Kaogu Yanjiusuo. Neimenggu Ningchengxian Xiaoyushulinzi Yizhi Shijue Jianban (The excavation of the Xiaoyushulinzi site at the county of Ningcheng in Inner Mongolia). Kaogu (Archaeology). 1965; 12: 619–621. In Chinese.

[30] Liaoningsheng Kaogu Yanjiusuo. Liaoning Jinzhou Xidalizi Yizhi Fajue Jianbao (The excavation of the Xidalizi site in Jinzhou, Liaoning). Beifang Minzu Kaogu (The Archaeology of Northern Ethnicity). 2015; 2: 1–15. In Chinese.

[31] Liaoningsheng Kaogu Yanjiusuo. Liaoning Lingyuanxian Sanguandianzi Chengzishan Yizhi Fajue Baogao (The excavation of the Chengzishan site at Sanguandianzi in the county of Liaoyuan, Liaoning). Kaogu (Archaeology). 1986; 6: 497–510. In Chinese.

[32] IA CASS. Chifeng Zhizhushan Yizhi de Fajue (The excavation of the Zhizhushan site in Chifeng). Kaogu Xuebao (Acta Archaeologica Sinica). 1979; 1: 111–144. In Chinese.

[33] IA CASS. Dadianzi: Xiajiadian Xiaceng Wenhua Yizhi yu Mudi Fajue Baogao (Dadianzi: The excavation of the cemetery of Lower Xiajiadian Culture). Beijing: Science Press; 1996. In Chinese.

[34] Liaoningsheng Kaogu Yanjiusuo. Liaoning Fuxin Pingdingshan Shichengzhi Fajue Baogao (The excavation of the Pingdingshan site in Fuxin, Liaoning). Kaogu (Archaeology). 1992; 5: 399–417. In Chinese.

[35] Neimenggu Wenwu Kaogu Yanjiusuo. Neimenggu Chifengshi Kangjiawan Yizhi 2006 Nian Fajue Jianbao (The excavation of the Kangjiawan site in 2006 in Chifeng, Inner Mongolia). Kaogu (Archaeology). 2008; 11: 15–23. In Chinese.

[36] Neimenggu Wenwu Kaogu Yanjiusuo. Chifengshi Songshanqu Sanzuodian Yizhi 2005 Niandu Fajue Baogao (The excavation of the Sanzuodian site in 2005 in the Songshan District, Chifeng). Neimenggu Wenwu Kaogu (Inner Mongolia Cultural Relic and Archaeology). 2006; 1: 1–8. In Chinese.

[37] Neimenggu Wenwu Kaogu Yanjiusuo. Neimenggu Chifengshi Sanzuodian Xiajiadian Xiaceng Wenhua Shicheng Yizhi (The site of Lower Xijiadian Culture at Sanzuodian in Chifeng, Inner Mongolia). Kaogu (Archaeology). 2007; 7: 17–27. In Chinese.

[38] Neimenggu Wenwu Kaogu Yanjiusuo. Chifeng Shangjiyingfangzi Xiliang Shichengzhi 2006 Nian Kaogu Fajue Jianbao (The excavation of the Xiliang site in 2016 at Shangjiyingfangzi in Chifeng). Bianjiang Kaogu Yanjiu (Research of China’s Frontier Archaeology). 2008; 6: 389–397. In Chinese.

[39] Neimenggu Wenwu Kaogu Yanjiusuo. Yantaishan Yizhi Fajue Jianbao (The excavation of the Yantaishan site). Nengmenggu Wenwu Kaogu (Inner Mongolia Cultural Relic and Archaeology). 2002; 2: 13–23. In Chinese.

[40] Bowuguan Wulanchabushi. Chifeng Songshanqu Shuidixiang Danangou Shicheng Diaocha Baogao (The survey report of the Danangou site at the county of Shuidi in Songshan District, Chifeng). Caoyuan Wenwu (Steppe Cultural Relics). 2015; 1: 53–56. In Chinese.

[41] Bak JH. Xiajiadian Xiaceng Wenhua Juluo, Jingji Yu Shehui Xingtai Yanjiu (A study of the settlement, economic and social patterns of the Lower Xiajiadian Culture). Ph. D. Dissertation, Graduate School at Chinese Academy of Social Sciences. 2020. In Chinese.

[42] Xu ZF. Shilun Xiajiadian Xiaceng Wenhua Shicheng (A discussion on the Stone-walled city of the Lower Xiajiadian Culture). Zhongyuan Wenwu (Cultural Relics of Central China). 2010; 3: 40–45. In Chinese.

[43] IA CASS. Banzhijianhe Zhongyou Xianqin Shiqi Yizhi (The archaeological site of pre-Qin period in the middle reaches of Banzhijian River). Beijing: Science Press; 2002. In Chinese.

[44] Wang LX. Shixi Xiajiadian Xiaceng Wenhua Yizhi de Leixing yu Buju Tedian (An analysis of the type and layout characteristics of the Lower Xiajiadian Culture sites). Wenwu Chunqiu. 2000; 3: 10–14. In Chinese.

[45] Teng MY, Ta L, Zhu YP, Guo ZZ, Shlach G., Drennan R., et al. Chifeng Diqu Juluo Fenbu de Yanbian (Changing Patterns of Settlement Distribution in the Chifeng Region). In: Chifeng Zhong-Mei Lianhe Kaogu Yanjiu Xiangmu (The Chifeng International Collaborative Archaeological Research Project), editor. Neimenggu Dongbu (Chifeng) Quyu Kaogu Diaocha Jieduanxing (Reginoal Archaeology in Eastern Inner Mongolia: A Methodological Exploration). Beijing: Science Press; 2003. pp.107–121. In Chinese.

[46] Zhao KL, Li XQ, Shang X. Qingtong Shidai Zhongwanqi Liaoxi Diqu Nongye Huodong Tezheng (Agricultural characteristics of middle-late Bronze Age in Western Liaoning Province). Zhiwu Xuebao (Chinese Bulletin of Botany). 2009; 44: 718–724. In Chinese.

[47] Sun YG. Shilun Xiajiadian Xiaceng Wenhua Shengye Fangshi — Yi Zhiwu Kaoguxue Wei Zhongxin (A discussion on the subsistence strategy of Lower Xiajiadian Culture — Based on archaeobotanical evidence). Neimenggu Shehuikexue (Inner Mongolia Social Sciences). 2013; 9: 45–48. In Chinese.

[48] Sun YG, Zhao ZJ, Cao JE, Sun JS, Dang Y. Neimenggu Erdaojingzi Yizhi 2009 Nian Fuxuan Jieguo Fenxi Baogao (The report of floatation results at the Erdaojingzi site in 2009, Inner Mongolia). Nongye Kaogu (Agricultural Archaeology). 2014; 6: 1–9. In Chinese.

[49] Li WY. Beipiao Kangjiatun Chengzhi ji Xiangguan Wenti Yanjiu (A study on the Kangjiatun site at Beipiao and related questions). Kaogu yu Wenwu (Archaeology and Cultural Relics). 2015; 1: 40–44. In Chinese.

[50] Lian JL. Neimenggu Chifeng Gaojiataizi Yizhi (The Gaojiataizi site at Chifeng, Inner Mongolia). Dazhong Kaogu (Public Archaeology). 2018; 12: 12–13. In Chinese.

[51] IA CASS. Zhongguo Kaoguxue zhong Tanshisi Niandai Shujuji 1965–1991 (The collection of 14C data in Chinese Archaeology 1965–1991). Beijing: Cultural Relics Publishing House; 1991. In Chinese.

[52] IA CASS. Fangshexing Tansu Niandai Ceding Baogao 24 (Radiocarbon dating report 24). Kaogu (Archaeology). 1997; 7: 35–38. In Chinese.

[53] IA CASS. Fangshexing Tansu Niandai Ceding Baogao 25 (Radiocarbon dating report 25). Kaogu (Archaeology). 1999; 7: 80–83. In Chinese.

[54] IA CASS. Fangshexing Tansu Niandai Ceding Baogao 26 (Radiocarbon dating report 26). Kaogu (Archaeology). 2000; 8: 70–74. In Chinese.

[55] IA CASS. Fangshexing Tansu Niandai Ceding Baogao 20 (Radiocarbon dating report 20). Kaogu (Archaeology). 1993; 7: 645–649. In Chinese.

[56] Bronk Ramsey C. Bayesian analysis of radiocarbon dates. Radiocarbon. 2009; 51: 337–360. 10.1017/S0033822200033865

[57] Reimer P, Austin W, Bard E, Bayliss A, Blackwell P, Bronk Ramsey C, et al. The IntCal20 Northern Hemisphere radiocarbon age calibration curve (0–55 cal kBP). Radiocarbon. 2020; 62: 725–757. 10.1017/RDC.2020.41

[58] Jones MK, Liu X. Origins of agriculture in East Asia. Sci. 2009 May 8; 324(5928): 730–1. doi: 10.1126/science.1172082 19423806

[59] Zhao ZJ. New Archaeobotanic Data for the Study of the Origins of Agriculture in China. Curr. Anthropol. 2011; 52: S295–306. 10.1086/659308

[60] Liu X, Jones MK, Zhao Z, Liu G, O’Connell TC. The earliest evidence of millet as a staple crop: New light on neolithic foodways in North China. Am. J. Phys. Anthropol. 2012 Oct;149(2): 283–90. doi: 10.1002/ajpa.22127 22961368

[61] Zhao ZJ. Cong Xinglonggou Yizhi Fuxuan Jieguo Tan Zhongguo Beifang Hanzuo Nongye Qiyuan (Addressing the Origins of Agriculture in North China Based on Results of Floatation from the Xinglonggou site). Dongya Guwu (Antiquities of Eastern Asia). 2004; 12: 188–199. In Chinese.

[62] Wang LX. Dashanqian Yizhi Fajue Ziliao suo Fanying de Xiajiadian Xiaceng Wenhua de Jingji Xingtai yu Huanjing Beijing (The economic form and environmental background of the Lower Xiajiadian Culture reflected in the excavation data of the Dashanqian site). Bianjiang Kaogu Yanjiu (Research of China’s Frontier Archaeology). 2007; 6: 350–357. In Chinese.

[63] Xiao XM, Tang ZW. Chifeng Shangjiyingfangzi Yizhi Rendi Guanxi Yanjiu (A study on human-land relationship at the Shangjiyingfangzi site in Chifeng). Caoyuan Wenwu (Steppe Cultural Relics). 2014; 1: 138–144. In Chinese.

[64] Li YY, Willis KJ, Zhou LP, Cui HT. The impact of ancient civilization on the northeastern Chinese landscape: palaeoecological evidence from the Western Liaohe River Basin, Inner Mongolia. The Holocene. 2006 Dec;16(8):1109–21. 10.1177/0959683606069403

[65] Kong ZC, Du NQ, Liu GM, Yang H. Neimenggu zizhiqu Chifengshi jujin 8000–2400 nianjian huanjing kaoguxue de chubu yanjiu (Preliminary Study on the Environment and Climate of the Chifeng Region during 8000–2400 BP). In: Zhou KS, Gong QM, editors. Huanjing Kaogu Yanjiu (Research on Environmental Archaeology). Beijing: Science Press; 1991. pp.112–119. In Chinese.

[66] Wang SZ, Wang ZL, Zhu YP. Neimenggu Chifengshi Dashanqi Diyi Didian Xiajiadian Xiaceng Wenhua de Zhibei he Shengtai Qihou (The vegetation and ecological climate of the first location at Dashanqian during the Lower Xiajiadian period, Chifeng, Inner Mongolia). Huaxia Kaogu (Huaxia Archaeology). 2004; 3: 44–51. In Chinese.

[67] Xiao J, Si B, Zhai D, Itoh S, Lomtatidze Z. Hydrology of Dali lake in central-eastern Inner Mongolia and Holocene East Asian monsoon variability. J Paleolimnol. 2008 Jul; 40(1): 519–28. 10.1007/s10933-007-9179-x

[68] Hunt HV, Campana MG, Lawes MC, PARK YJ, Bower MA, Howe CJ, et al. Genetic diversity and phylogeography of broomcorn millet (Panicum miliaceum L.) across Eurasia. Molecular ecology. 2011 Nov; 20(22): 4756–71. doi: 10.1111/j.1365-294X.2011.05318.x 22004244 PMC3258423

[69] Yang X, Wu W, Perry L, Ma Z, Bar-Yosef O, Cohen DJ, et al. Critical role of climate change in plant selection and millet domestication in North China. Sci Rep. 2018 May 18; 8(1):1–9. doi: 10.1038/s41598-018-26218-6 29777204 PMC5959876

[70] Shelach-Lavi G, Raphael K, Jaffe Y. Sanzuodian: The Structure, Function and Social Significance of the Earliest Stone Fortified Sites in China. Antiquity. 2011; 85: 11–26. doi: 10.1017/S0003598X00067405

[71] Shelach-Lavi G. The Archaeology of Ancient China: From Prehistory to the Han Dynasty. Cambridge: Cambridge University Press; 2015. pp.132–139.

[72] Zhang XD, Xin Y. Da, Xiaolinghe liuyu Xiajiadian xiaceng wenhua juluo de chubu renshi (A Preliminary Study on the settlement of Lower Xiajiadian Culture in the Daling and Xiaoling River Valleys). Dongfang Kaogu (Archaeology of Eastern China). 2015; 11: 93–102. In Chinese.

[73] Cui Y, Li H, Ning C, Zhang Y, Chen L, Zhao X, et al. Chromosome analysis of prehistoric human populations in the West Liao River Valley, Northeast China. BMC Evol Biol. 2013 Dec;13(1):1–10. doi: 10.1186/1471-2148-13-216 24079706 PMC3850526

[74] Li H, Zhao X, Zhao Y, Li C, Si D, Zhou H, et al. Genetic characteristics and migration history of a bronze culture population in the West Liao-River valley revealed by ancient DNA. J Hum Genet. 2011 Dec; 56(12): 815–22. doi: 10.1038/jhg.2011.102 21938002

[75] Ning C, Li T, Wang K, Zhang F, Li T, Wu X, et al. Ancient genomes from northern China suggest links between subsistence changes and human migration. Nat. Commun. 2020 Jun 1;11(1):1–9. doi: 10.1038/s41467-020-16557-2 32483115 PMC7264253

[76] Chen GQ, Zhang QC. Yinhe, Yingjinhe liuyu Xiajiadian xiaceng wenhua shichengzhi yanjiu (A Study on Stone-Walled Settlements of Lower Xiajiadian Culture in the Yin River and Yinjing River Valleys). Shehui Kexue Zhanxian (Social Science Front). 2012; 5: 106–111. In Chinese.

[77] Xia BG. Liaoxi Xiajiadian xiaceng wenhua juluo de fangyuxing ji xiangguan wenti (The defensiveness and related problems of settlements of Lower Xiajiadian Culture in Liaoxi area). Beifang Wenwu (Northern Cultural Relics). 2011; 4: 35–40. In Chinese.

[78] Zheng SZ. Hebei Pingquan yidai faxian de shicheng juluo yizhi—jian lun Xiajiadian xiaceng wenhua de chengbaodai wenti (The stone-walled settlements in Pingquan, Hebei: A discussion on settlements of Lower Xiajiadian Culture). Wenwu Chunqiu. 2003; 4: 1–6. In Chinese.

[79] Sun YG. Liaoxi diqu xinshiqi shidai zhi qingtong shidai kaoguxue wenhua yanjiu lunshu (A discussion on the research of archaeological cultures from Neolithic to Bronze Age in Western Liaoning). Chifeng Xueyuan Xuebao (Journal of Chifeng University). 2007; 5: 4–6. In Chinese.

[80] Han JY. Shilun zuowei changcheng yuanxing de beifang zaoqi shicheng dai (The belt of stone forts as the prototype for the historical long walls). Huaxia Kaogu (Huaxia Archaeology). 2008; 1: 48–59. In Chinese.

[81] Hubeisheng Wenwu Kaogu Yanjiusuo. Panlongcheng: 1963–1994 Nian Kaogu Fajue Baogao (Panlongcheng: The Report of Excvation From 1963 to 1994). Beijing: Cultural Relics Publishing House; 2001. In Chinese.

[82] Shandong University, Shandongsheng Wenwu Kaogu Yanjiusuo, Jinanshi Kaogu Yanjiusuo. Jinanshi Daxinzhuang Shangdai Juzhi he Muzang (The settlement and burials of Shang Dynasty at the Daxinzhuang site, Jinan). Kaogu (Archaeology). 2004; 7: 25–33. In Chinese.

[83] Liu SE. Laoniupo: Xibei Daxue Kaogu Zhuanye Tianye Fajue Baogao (Laoniupo: The excavation report of Archaeology Department at Northwestern Universiy). Xi’an: Shaanxi People’s Publishing House; 2002. In Chinese.

[84] Beijingshi Wenwu Guanlichu. Beijingshi Pingguxian Faxian Shangdai Muzang (The burials of Shang Dynasty at Pinggu, Beijing). Wenwu (Cultural Relics). 1977; 11: 1–8. In Chinese.

[85] Tianjingshi Wenhuaju Kaogu Fajuedui. Hebei Dachang Huizui Zizhixian Datuotou Yizhi Shijue Jianbao (The excavation report of the Datuotou site at Dachang Hui Autonomous County, Hebei). Kaogu (Archaeology). 1966; 1: 8–13. In Chinese

[86] Tianjinshi Wenwu Guanli Chu. Tianjin Jixian Zhangjiayuan Yizhi Shijue Jianbao (The excavation report of the Zhangjiayuan site at Ji County, Tianjin). Wenwu Ziliao Congkan (Cultural Relics Series). 1977; 1: 163–171. In Chinese.

[87] Tianjinshi Lishibowuguan Kaogudui. Tianjin Jixian Zhangjiayuan Yizhi Dierci Fajue (The second excavation of the Zhangjiayuan site at Ji County, Tianjin). Kaogu (Archaeology). 1984; 8: 698–705. In Chinese.

[88] Tianjin Lishibowuguan Kaogubu. Tianjin Jixian Zhangjiayuan Yizhi Disanci Fajue (The third excavation of the Zhangjiayuan site at Ji County, Tianjin). Kaogu (Archaeology). 1993; 4: 311–323. In Chinese.

[89] Beijingshi Wenwu Yanjiusuo. Changping Zhangying: Yanshan Nanlu Diqu Zaoqi Qingtong Wenhua Yizhi Fajue Baogao (Changping Zhangying: The excavation report of Early Bronze Age sites in the southern foot of Yan Mountains). Beijing: Cultural Relics Publishing House; 2007. In Chinese.

[90] Yang JH. Shilun Xiashang Shiqi Yanshan Yinan Diqu de Wenhua Geju (A Preliminary Study of the Culture Pattern on the South of Yan Mountains in the Xia and Shang Dynasties). Beifang Wenwu (Northern Cultural Relics). 1999; 3: 1–9. In Chinese.

[91] Yue LJ. Shangdai Bianyuan Diqu Erligang Wenhua Fenxi — Jianlun Shangdai Zaoqi de Zhengzhi Jiangyu (An Analysis of the Erligang Culture in the Frontier Regions of the Shang Dynasty — The Political Territory of the early Shang Dynasty). Kaogu Yu Wenwu (Archaeology and Cultural Relics). 1993; 4: 58–69. In Chinese.

[92] Bagley R. Shang Archaeology. In: Loewe M, Shaughnessy EL, editors. The Cambridge history of ancient China: From the origins of civilization to 221 BC. New York: Cambridge University Press; 1999. pp. 123–231.

[93] Wang HC. China’s first empire? Interpreting the material record of the Erligang expansion. In: Steinke K, Ching CY, editors. Art and archaeology of the Erligang civilization. Princeton: P.Y. and Kinmay W. Tang Center for East Asian Art, Department of Art and Archaeology, Princeton University: in Association with Princeton University Press; 2014. pp.67–97.

[94] Campbell R. Erligang in regional and diachronic context. In: Steinke K, Ching CY, editors. Art and archaeology of the Erligang civilization. Princeton: P.Y. and Kinmay W. Tang Center for East Asian Art, Department of Art and Archaeology, Princeton University: in Association with Princeton University Press; 2014. pp.121–135.

[95] Campbell R. Toward a Networks and Boundaries Approach to Early Complex Polities. Current Anthropology. 2009; 50 (6): 821–48.

[96] Zou H. Xishangzhou Kaogu Lunwenji (Collected Archaeological Papers of Xia, Shang, and Zhou). Beijing: Cultural Relics Publishing House; 1980. In Chinese.

[97] Zhang ZP, Kong ZS, Zhang WJ, Chen Y. Xiajiadian xiaceng wenhua yanjiu (Research of the Lower Xiajiadian culture). In: Su BQ, editor. Kaoguxue wenhua lunji (Collected papers of archaeological culture). Beijing: Culture Relics Publishing House; 1987. pp. 58–78.

[98] Weneuju Guojia. Zhongguo Wenwu Dituji: Shaanxi Fence (Atlas of Chinese Cultural Relics: The Volume of Shaanxi). Xi’an: Xi’an Cartographic Publishing House; 1998. In Chinese.

[99] Shaanxisheng Kaogu Yanjiuyuan. Shaanxi Shenmu Xian Shimao Yizhi (The Shimao site at Shenmu County). Kaogu (Archaeology). 2013; 7: 15–24.

[100] Shao J. Shilun Shimao Chengzhi de Niandai ji Xiujian Guocheng (A discussion on the age and construction of the Shimao site). Kaogu yu Wenwu (Archaeology and Cultural Relics). 2016; 4: 102–108.

[101] Jaffe Y, Campbell R, and Shelach-Lavi G. Shimao and the Rise of States in China. Current Anthropology. 2022; 63 (1): 95–117. 10.1086/719398.

[102] Gotlib AI, Podol’skii ML. Sve-gornye sooruzheniia Minusinskoy kotloviny (Sve-Mountain constructions of the Minusinsk Valley). Saint Petersburg: Elexis-Print; 2008. In Russian.

[103] Neimenggu Wenwu Kaogu Yanjiusuo. Daihai Kaogu (1): Laohushan Wenhua Yizhi Fajue Baogao (Archeology of the Daihai Region, Vol.1: The excavation of sites associated with the Laohushan Culture). Beijing: Science Press; 2000. In Chinese.

[104] Han JY. Laohushan Wenhua de Kuozhang yu Duiwai Yingxiang (A discussion on the expansion and influence of the Laohushan Culture). Zhongyuan Wenwu (Cultrual Relics of Central China). 2007; 1: 20–26. In Chinese.

