# Supplementary material for: From rammed earth to stone wall: Chronological insight into the settlement change of the Lower Xiajiadian culture
Source: PLoS One. 2022 Aug 22;17(8):e0273161. doi: 10.1371/journal.pone.0273161 (PMC9394813; doi:10.1371/journal.pone.0273161)
Supplement: S1 Table — (DOCX) [file pone.0273161.s001.docx]

Table S1. Radiocarbon dates for the Lower Xiajiadian Culture.

| Site | Lab No. | Material | Archaeological Context | Radiocarbon Age (bp) | Cal BC (68.3%) | Cal BC (95.4%) | References |
| --- | --- | --- | --- | --- | --- | --- | --- |
| Zhizhushan,  Inner Mongolia  (42°20′N, 118°57′E) | ZK-0176 | Charcoal | H42 | 3965±90 | 2580-2299 | 2856-2201 | IA CASS 1991: 55. |
| Shuiquan, Jianping, Liaoning  (41°17′25.34″N, 119°54′36.77″E) | ZK-0699 | Charcoal | T15(5) | 3780±90 | 2301-2029 | 2457-1950 | IA CASS 1991: 66. |
|  | ZK-0695 | Charcoal | T26(4) F41 | 3540±75 | 2019-1774 | 2136-1688 | “ |
| Redianchang, Chaoyang, Liaoning  (41°35′0.00″N, 120°27′0.00″E) | ZK-2222 | Charcoal | T13(2) | 3535±55 | 1956-1777 | 2025-1746 | IA CASS 1991: 67. |
|  | ZK-2223 | Charcoal | T6(3) | 3430±250 | 2043-1739 | 2278-1462 | IA CASS 1991: 68. |
|  | ZK-2224 | Charcoal | T6(4) | 3580±75 | 2027-1823 | 2131-1746 | “ |
|  | ZK-2225 | Charcoal | T6(4) F20 | 3725±135 | 2127-1822 | 2345-1743 | “ |
| Fengxia, Beipiao, Liaoning  (41°54′N, 120°37′E) | ZK-0153 | Charcoal | T9, 10(3) | 3550±80 | 2019-1767 | 2136-1645 | “ |
| Fanzhangzi, Aohan,  Inner Mongolia  (42°17′N, 119°55′E) | WB82-38 | Wooden  remains | Tomb M78 | 3180±90 | 1614-1325 | 1741-1235 | IA CASS 1991: 56. |
|  | ZK-1208 | Wooden remains | Tomb M79 | 3545±95 | 1972-1696 | 2133-1619 | “ |
|  | ZK-1209 | Wooden remains | Tomb M84 | 3510±75 | 1919-1696 | 2023-1624 | “ |
| Dadianzi, Aohan,  Inner Mongolia  (42°18′N, 120°36′E) | ZK-0402 | Wooden remains | Tomb M454 | 3390±90 | 1866-1550 | 1899-1501 | “ |
|  | ZK0480 | Wooden  remains | Tomb M759 | 3420±85 | 1872-1616 | 1919-1516 | “ |
| Dashanqian  Harqin, Inner Mongolia  (41°54′N, 118°36′E) | ZK-2934 | Charcoal | 96KD I H12 | 3834±400 | 2877-1775 | 3498-1314 | IA CASS 1997: 35. |
|  | ZK-2935 | Charcoal | 96KD I H34 | 3157±75 | 1514-1384 | 1606-1286 | “ |
|  | ZK-2938 | Charcoal | 96KD I T111(2) | 3418±70 | 1721-1537 | 1819-1505 | “ |
|  | ZK-2939 | Charcoal | 96KD I T408(4) | 3403±83 | 1696-1531 | 1820-1455 | IA CASS 1999: 80. |
|  | ZK-2941 | Charcoal | 96KD I T434 F18 | 3462±79 | 1761-1541 | 1866-1512 | “ |
|  | ZK-3017 | Charcoal | 98KD IV M1 | 3141±51 | 1498-1390 | 1509-1301 | IA CASS 2000: 70. |
|  | ZK-3025 | Charcoal | 98KD IV H210(3) | 3374±55 | 1683-1537 | 1746-1507 | IA CASS 2000: 71. |
|  | ZK-3032 | Charcoal | 98KD IV H246(4) | 3140±56 | 1498-1389 | 1516-1291 | “ |
|  | ZK-3033 | Charcoal | 98KD IV H306 | 3180±57 | 1506-1415 | 1603-1308 | “ |
|  | ZK-3034 | Charcoal | 98KD IV H294(1) | 3027±55 | 1412-1275 | 1442-1166 | “ |
|  | ZK-3035 | Charcoal | 98KD IV F62(2) | 3164±57 | 1502-1401 | 1538-1302 | “ |
|  | ZK-3036 | Charcoal | 98KD IV F66(2) | 3184±77 | 1533-1404 | 1616-1305 | “ |
| Location 342, Chifeng,  Inner Mongolia  (42°2′54.67″N, 118°45′9.14″E) | BA-6769 | Charcoal | 054(4) | 3190±35 | 1510-1453 | 1599-1417 | Chifeng International Collaborative Archaeological Research Project 2011: 21. |
|  | BA-6770 | Charcoal | 055(5) | 3335±35 | 1616-1536 | 1669-1513 | “. |
|  | BA-6771 | Charcoal | 093(7) | 3235±35 | 1541-1463 | 1610-1447 | “ |
|  | BA-6772 | Charcoal | 271(24) | 3280±35 | 1601-1511 | 1621-1497 | “ |
|  | BA-6773 | Charcoal | 309(13) | 3375±40 | 1654-1538 | 1697-1520 | “ |
|  | BA-6774 | Charcoal | 420(15) | 3370±35 | 1653-1539 | 1695-1529 | “ |
|  | BA-6775 | Charcoal | 426(16) | 3370±35 | 1653-1540 | 1695-1529 | “ |
|  | BA-6776 | Charcoal | 578(26) | 3260±35 | 1601-1500 | 1616-1459 | “ |
|  | BA-6777 | Charcoal | 584(26) | 3300±35 | 1600-1529 | 1622-1505 | “ |
|  | BA-6778 | Charcoal | 585(24) | 3305±35 | 1603-1531 | 1626-1505 | “ |
| Location 674/Dianjiangtai, Chifeng,  Inner Mongolia  (42°12’N, 118°54’E) | BA-2241 | Charcoal | 674X054 | 3183±60 | 1518-1442 | 1598-1389 | “ |
|  | BA-2242 | Charcoal | 674X052 | 3230±60 | 1532-1454 | 1600-1420 | “ |
|  | BA-2243 | Charcoal | 674X049 | 3100±100 | 1531-1424 | 1601-1310 | “ |
|  | BA-2244 | Charcoal | 674X047 | 3220±60 | 1530-1451 | 1600-1416 | “ |
|  | ZK-2651 | Charcoal | 90MCDT103(13) | 3278±55 | 1551-1455 | 1616-1438 | IA CASS 1993: 646. |
|  | ZK-2652 | Charcoal | 90MCDT104(6) | 3327±58 | 1579-1461 | 1662-1441 | “ |
| Sanzuodian, Chifeng,  Inner Mongolia  (42°21′47.49″N, 118°36′47.08″E) | BA-6837 | - | - | 3025±35 | 1386-1231 | 1412-1197 | Chifeng International Collaborative Archaeological Research Project 2011: 21. |
|  | BA-6838 | - | - | 3240±30 | 1531-1452 | 1604-1429 | “ |
|  | BA-6839 | - | - | 3265±30 | 1600-1460 | 1612-1451 | “ |
